# Neuromodulation of inhibitory synaptic transmission in the basolateral amygdala during fear and anxiety

**DOI:** 10.3389/fncel.2024.1421617

**Published:** 2024-06-27

**Authors:** Xin Fu, Jeffrey G. Tasker

**Affiliations:** ^1^McGovern Institute for Brain Research, Massachusetts Institute of Technology, Cambridge, MA, United States; ^2^Department of Brain and Cognitive Sciences, Massachusetts Institute of Technology, Cambridge, MA, United States; ^3^Tulane Brain Institute, Tulane University, New Orleans, LA, United States; ^4^Department of Cell and Molecular Biology, Tulane University, New Orleans, LA, United States

**Keywords:** interneuron, GABA, norepinephrine, basket cell, parvalbumin, CCK, memory, oscillation

## Abstract

The basolateral amygdala plays pivotal roles in the regulation of fear and anxiety and these processes are profoundly modulated by different neuromodulatory systems that are recruited during emotional arousal. Recent studies suggest activities of BLA interneurons and inhibitory synaptic transmission in BLA principal cells are regulated by neuromodulators to influence the output and oscillatory network states of the BLA, and ultimately the behavioral expression of fear and anxiety. In this review, we first summarize a cellular mechanism of stress-induced anxiogenesis mediated by the interaction of glucocorticoid and endocannabinoid signaling at inhibitory synapses in the BLA. Then we discuss cell type-specific activity patterns induced by neuromodulators converging on the Gq signaling pathway in BLA perisomatic parvalbumin-expressing (PV) and cholecystokinin-expressing (CCK) basket cells and their effects on BLA network oscillations and fear learning.

## 1 Introduction

Anxiety and fear-related disorders, characterized by maladaptive regulation of fear and excessive and unrestrained anxiety, are highly prevalent and debilitating mental illnesses with a life-time prevalence of ~14% around the world and billions of dollars of socio-economic burden (Kessler et al., 2009; Ressler, 2020). A deeper understanding of the cellular mechanisms underlying fear and anxiety disorders is required to develop better treatments. Among a broad and interconnected network of brain areas controlling fear and anxiety, the basolateral amygdala (BLA) plays a pivotal role in the regulation of both fear and anxiety, which are profoundly regulated by multiple neuromodulators, including norepinephrine, serotonin, acetylcholine, and endocannabinoids (Minor and Hunter, 2002; Lutz et al., 2015; Tovote et al., 2015; Giustino and Maren, 2018; Likhtik and Johansen, 2019). During emotional arousal, combinations of these neuromodulators act on their respective G protein-coupled receptors (GPCRs) to regulate the neuronal activity and network state of the BLA and ultimately the behavioral expression of fear and anxiety.

The BLA is a cortex-like structure in the limbic system based on its cellular composition. While the majority of the BLA neurons are glutamatergic principal cells orchestrating brain-wide responses through long-range projections, a smaller percentage of BLA neurons is comprised of highly diverse GABAergic inhibitory interneurons that tightly control the electrical activity of the principal cells in a temporally and spatially specific manner to regulate synaptic integration and shape synchronized oscillations of the BLA neural network (Wolff et al., 2014; Tovote et al., 2015; Davis et al., 2017; Krabbe et al., 2018; Fu et al., 2022). Based on their postsynaptic targets, BLA interneurons can be categorized into three broad types: (1) perisomatic inhibitory interneurons, including the cholecystokinin (CCK)-positive and parvalbumin (PV)-positive basket cells and axo-axonic cells, which selectively innervate the soma, proximal dendrites, and axonal initial segment of BLA principal neurons to effectively control spike timing; (2) dendritic inhibitory interneurons, comprised mainly of the somatostatin (SOM)-positive interneurons, which primarily synapse on the dendrites of principal cells to regulate synaptic integration; and (3) interneuron-selective interneurons, like the vasoactive intestinal peptide (VIP)-positive interneurons, which selectively target other interneurons to disinhibit the principal neurons. There are also SOM-positive inhibitory neurons in the BLA that are considered projection neurons rather than interneurons since they send long-range axons to other brain areas that communicate with the BLA (McDonald et al., 2012; McDonald and Zaric, 2015), and neurogliaform cells that are thought to signal mainly through volume transmission (Oláh et al., 2009; Mańko et al., 2012).

With the aid of cell type-specific recordings and manipulations, a large body of knowledge has contributed to our understanding of the role of different interneurons in the regulation of fear conditioning, which has been summarized in detail earlier (Ehrlich et al., 2009; Krabbe et al., 2018; Hájos, 2021). Accumulating evidence from recent studies also reveals that neuromodulation of these interneurons and inhibitory neurotransmission in the BLA profoundly impacts anxiety and fear learning. Recent studies from our group on neuromodulation of inhibitory synaptic transmission have revealed (1) endocannabinoid modulation of synaptic inhibition in the BLA that mediates acute anxiogenic actions of stress hormones, and (2) convergent actions of multiple neuromodulators on Gq G-protein signaling pathways to generate distinct stereotyped patterned outputs from the perisomatic basket cells that regulate fear expression. These studies reveal novel working principles of neuromodulatory systems and expand our understanding of how neuromodulation of inhibitory synaptic transmission in the BLA regulates fear and anxiety.

## 2 Stress-induced anxiogenesis mediated by endocannabinoid suppression of inhibition

### 2.1 Regulation of anxiety by synaptic inhibition in the BLA

Extensive human and animal studies have shown that hyperexcitability of the BLA promotes anxiety and anxiety-like behaviors through its cascade of connections with other fear-related structures including the central amygdala (CeA) and the bed nucleus of the stria terminalis (BNST) (Ressler, 2010; Tovote et al., 2015; Babaev et al., 2018). Although local inhibitory GABAergic interneurons only account for 22% of the total BLA neuron population (Viktória et al., 2021), a change in the level of inhibition in the BLA shifts the excitation-inhibition balance, leading to either hypo- or hyperactivity of the BLA and the subsequent increase or decrease in anxiety. It has been shown that acute ethanol in the BLA induces a PV interneuron-dependent increase in inhibitory synaptic transmission in BLA principal cells and reduces anxiety-like behaviors in male but not female rats (Soumyabrata et al., 2023). Moreover, rats that experience trauma-like high-intensity shock display desensitization to norepinephrine and serotonin facilitation of inhibitory synaptic transmission in the BLA (Braga et al., 2004; Jiang et al., 2009). Additionally, post-weaning isolation in juvenile rats, a model that increases anxiety levels in adulthood (Lukkes et al., 2009), leads to attenuated c-Fos expression in BLA PV interneurons compared with group-reared rats (Lukkes et al., 2012). These findings collectively indicate that inhibitory synaptic transmission in the BLA plays an important role in the regulation of anxiety-like behavior and suggest that dysregulated inhibition in the BLA may provide a neural mechanism of pathological anxiogenesis.

### 2.2 Endocannabinoid signaling in the BLA

Distinct from classical neurotransmitters (e.g., glutamate and GABA) and neuromodulators (e.g., norepinephrine, acetylcholine, and serotonin), endocannabinoids (eCBs), including anandamide (AEA) and 2-arachidonoylglycerol (2-AG), are not stored in synaptic vesicles but are synthesized and released on demand (Freund et al., 2003; Marsicano and Lutz, 2006). In the brain, released endocannabinoids travel retrogradely to bind to the type 1 cannabinoid (CB1) receptor on presynaptic terminals. CB1 receptors are typically coupled to the Gi/o signaling pathway and once activated, suppress the release of neurotransmitters at synapses via inhibition of calcium influx through voltage-gated calcium channels and suppression of the cAMP-PKA signaling pathway (Brown et al., 2003; Castillo et al., 2012). In the BLA, CB1 receptors are found to be expressed on glutamatergic terminals at a low level and on the GABAergic terminals of CCK basket cells at a high level (Katona et al., 2001; Yoshida et al., 2011). Interestingly, even though the CB1 receptors have similar efficacy at CCK basket cell synapses on BLA principal neurons projecting to the prelimbic (PL-PFC) and the infralimbic prefrontal cortex (IL-PFC), the higher level of expression of the 2-AG synthetic enzyme, diacylglycerol lipase α, in the IL-PFC-projecting BLA principal neurons endows pathway-specific regulation of the CCK basket cell-mediated inhibition, which may boost the activity of the BLA-to-IL-PFC pathway and facilitate extinction learning (Vogel et al., 2016). Depending on the type of synapse, eCB’s can either increase the excitability of BLA principal cells by suppressing GABA release or decrease excitability by inhibiting glutamate release. Consistent with the idea that general hyperactivity of the BLA promotes anxiety, forebrain deletion of CB1 receptors at GABA synapses decreases and deletion of CB1 receptors at glutamate synapses increases anxiety-like behaviors (Lafenêtre et al., 2009; Häring et al., 2011; Petrie et al., 2021), although the effect of knockout of CB1 receptors specifically in the BLA needs to be further tested.

Recent technical advances in circuit mapping, manipulation, and imaging have greatly facilitated our understanding of pathway-specific eCB modulation of BLA function in fear and anxiety. The principal neurons in the BLA are functionally heterogeneous and their roles can largely be defined by their projection targets. Accumulating evidence has revealed that the reciprocal connections between the BLA and the IL-PFC are critical for the extinction of fear memories, while the connections between the BLA and the PL-PFC promote sustained fear and anxiety-like behavior (Quirk and Mueller, 2008; Burgos-Robles et al., 2009, 2017; Sierra-Mercado et al., 2011; Felix-Ortiz et al., 2016). Consistent with this, a recent elegant study by Marcus et al. (2020) showed that exposure to acute stress across multiple modalities induced a persistent reduction in 2-AG-mediated suppression of excitatory synaptic transmission in the BLA-to-PL-PFC synapses, which caused stress-induced strengthening of the BLA-PL-PFC reciprocal circuit activity. Moreover, selective deletion of DAGLα in the PL-PFC or CB1 receptors in the PL-PFC-projecting BLA neurons augmented stress-induced anxiety (Marcus et al., 2020). In addition to the modulation of BLA synapses in the PL during stress exposure, eCBs are also recruited by optogenetic activation of the IL projections in the BLA, which facilitates fear memory extinction (Gunduz-Cinar et al., 2023). Intriguingly, using imaging of eCB dynamics with an eCB GRAB sensor in IL-PFC axon terminals in the BLA, it was shown that the eCBs are selectively mobilized at the offset of shock-associated cues, especially at the early phase of extinction training, which gradually subsides with repeated cue presentations in the later phase of extinction training. This suggests that high eCBs at IL-BLA synapses may encode unexpected shock omission at early extinction while a decreased level of eCBs after extinction training may disinhibit glutamatergic transmission between IL and BLA to promote extinction learning (Gunduz-Cinar et al., 2023).

### 2.3 Stress-induced suppression of inhibition promotes anxiety

Experimentally, endocannabinoids, particularly 2-AG, can be mobilized by strong depolarization of the postsynaptic neurons in brain slices to suppress the release of glutamate or GABA, a phenomenon known as depolarization-induced suppression of excitation (DSE) or inhibition (DSI), respectively (Wilson et al., 2001; Wilson and Nicoll, 2001; Castillo et al., 2012). It was recently shown *in vivo* that discharges of place cells in the hippocampal CA1 region also trigger 2-AG release, which suppresses CCK basket cell-mediated inhibition (Dudok et al., 2024). Under physiological conditions, stress, including acute restraint for 30 min, reliably elevates the level of 2-AG in the BLA in rodents (Patel et al., 2005; Hill and McEwen, 2009; Hill and Tasker, 2012). The stress-induced increase in 2-AG in the BLA shows a delayed time course that correlates to the rise in circulating glucocorticoids, suggesting an interaction between glucocorticoids and endocannabinoids (Hill et al., 2011; Petrie et al., 2021). Supporting this idea, it was shown that application of corticosterone or the synthetic glucocorticoid analog dexamethasone rapidly and reliably induced a long-lasting decrease in the frequency of miniature inhibitory postsynaptic currents (mIPSCs) recorded in BLA principal cells in brain slices, the effect of which was blocked by pre-application of the CB1 receptor antagonist SR141716 (Di et al., 2016). Given that the CB1 receptors are predominantly expressed in the axon terminals of CCK basket cells, it is likely that glucocorticoid-induced eCB synthesis modulates synaptic GABA transmission at CCK basket cell axons. Similar to glucocorticoid-induced eCB actions in the hypothalamus (Di et al., 2003; Tasker et al., 2006), this rapid effect is mediated by activation of a membrane-associated glucocorticoid receptor that signals via a G-protein signaling cascade, as it was blunted by blocking G-protein signaling and mimicked by bath application of a membrane-impermeant dexamethasone conjugate, but not by intracellular application of dexamethasone (Di et al., 2016). The dampened synaptic inhibition in BLA principal cells by glucocorticoid-mobilized eCB shifts the excitation-inhibition balance toward greater excitation, leading to a pro-anxiety state. Consistent with this, intra-BLA infusion of the CB1 receptor antagonist SR141716 or the 2-AG synthesis inhibitor tetrahydrolipstatin attenuated the stress-induced decrease in time in the center in an open field test (Di et al., 2016). Similarly, reducing the anandamide level in the BLA by overexpression of fatty acid amide hydrolase led to reduced anxiety-like behavior in male rats via a GABA_A_ receptor-dependent mechanism, supporting an endocannabinoid modulatory role at inhibitory synapses in anxiety-like behaviors (Morena et al., 2019). Overall, neuromodulatory suppression of inhibition by functional interaction between glucocorticoids and endocannabinoids in the BLA provides a cellular mechanism for stress-related anxiogenesis.

## 3 Neuromodulatory regulation of PV and CCK inhibitory interneurons drives distinct neural activity patterns and oscillatory states in the BLA

### 3.1 Multiple neuromodulators are released in the BLA during emotional arousal

It is well established that the process of fear regulation is tightly controlled by neuromodulators including norepinephrine (NE), acetylcholine, and serotonin that carry information about emotional arousal and attention (McGaugh, 2004; Lee and Dan, 2012; Likhtik and Johansen, 2019). Anatomically, the BLA receives dense innervation by subcortical noradrenergic, serotonergic, and cholinergic afferents, which provides a circuit basis for the neuromodulation of BLA function (Kitt et al., 1994; Muller et al., 2007; Zhang et al., 2013; Schwarz et al., 2015; McCall et al., 2017; Crimmins et al., 2023). During stressful experiences, NE, acetylcholine, and serotonin are released in the BLA and a mixture of these neuromodulators bind to their cognate receptors on excitatory and inhibitory neurons to alter the neural activity and network state of the BLA (Rueter and Jacobs, 1996; Amat et al., 1998; McIntyre et al., 2002; Likhtik and Johansen, 2019; Kellis et al., 2020; Totty and Maren, 2022). In this section, we focus on the binding of these neuromodulators to their G protein-coupled receptors that leads to Gq neuromodulatory activation of BLA interneurons and we discuss the effect of this neuromodulation on BLA neural output and fear processing.

The Gq G protein comprises one of the four G-protein families of Gα subunits that form heterotrimeric G proteins with Gβ and Gγ subunits and interact with G protein-coupled receptors to transduce extracellular signals into intracellular signaling cascades (Kamato et al., 2015; Campbell and Smrcka, 2018). Conventionally, liganded Gq-coupled GPCRs activate phospholipase C to cleave phosphatidylinositol 4,5-bisphosphate into inositol 1,4,5-trisphosphate and diacylglycerol, which leads to an increase in intracellular [Ca^2+^], activation of protein kinase C, and, frequently, ion channel modulation and neuron activation (Huang et al., 2007; Kamato et al., 2015; Zhang et al., 2022). Recent discovery of the specific Gq protein inhibitors, YM-254890 and FR900359, which block the exchange of Gq from the inactive GDP-bound to the active GTP-bound state (Nishimura et al., 2010; Schrage et al., 2015), has greatly facilitated the study of Gq signaling in numerous cellular processes (Schrage et al., 2015; Kamato et al., 2017; Kostenis et al., 2020; Voss, 2023). In addition, the Gq-coupled designer receptor hM3D has been used extensively and reliably in neuroscience research to activate specific populations of neurons and to mimic the excitatory effect of native Gq-coupled G protein-coupled receptors, like the type 1 metabotropic glutamate receptors (Roth, 2016; Atasoy and Sternson, 2017). For a neuromodulator-activated neural response to be considered as Gq-dependent, minimum requirements are that (1) the response is completely blocked by a Gq inhibitor and (2) it is mimicked by activation of the exogenously expressed Gq-coupled designer receptor, hM3D.

### 3.2 Functional dichotomy of PV- and CCK-positive basket cells

Both PV-positive and CCK-positive basket cells selectively innervate and inhibit the perisomatic regions of hundreds of principal neurons to control the spike timing and synchronization of population neural activity (Freund and Katona, 2007; Vereczki et al., 2016; Veres et al., 2017). Yet, these two types of basket cells show very distinct properties in their biochemical compositions, synaptic connectivity, and involvement in neural oscillations (Freund and Katona, 2007; Armstrong and Soltesz, 2012). At synaptic terminals, P/Q-type calcium channels are activated in PV basket cells, whereas N-type calcium channels are activated in CCK basket cells to stimulate vesicular GABA release. Vesicular GABA release is strongly modulated by endocannabinoids in CCK basket cell terminals, but not in PV basket cell terminals, due to the high expression of CB1 receptors in CCK neurons, but not in PV neurons (Katona et al., 2001; Wilson et al., 2001; Yoshida et al., 2011; Rovira-Esteban et al., 2019). By virtue of abundant and selective expression of CB1 receptors on their axon terminals, CCK basket cells provide another level of modulation via DSI and G protein-coupled receptor-mediated eCB mobilization to sculpt circuit activity (Di et al., 2016; Dudok et al., 2024). At the circuit level, the PV basket cells in the BLA receive much denser glutamatergic input than the CCK basket cells, resulting in a more reliable activation by synaptic stimulation of the PV basket cells than of the CCK basket cells (Andrási et al., 2017). Field potential recordings of synchronous population activity in the hippocampus show that the CCK basket cells are more strongly coupled to theta oscillations, while the PV basket cells are more coupled to gamma oscillations (Hájos et al., 2004; Klausberger et al., 2005; Bartos et al., 2007). Additionally, PV cells were shown to be both necessary and sufficient for the generation of gamma oscillations as optogenetic activation and inhibition of PV cells induced and suppressed gamma oscillations, respectively (Cardin et al., 2009; Sohal et al., 2009). With a novel transgenic mouse line that selectively labeled CCK basket cells in the hippocampal CA1 region, Dudok et al. (2021) revealed that CCK and PV basket cells displayed opposing activities during spontaneous behaviors, with PV basket cells active and CCK basket cells inhibited when mice were locomotive, a pattern that was reversed when the mice were immobile. Moreover, recent studies have further demonstrated the functional dichotomy of the PV and CCK basket cells upon Gq neuromodulatory activation, which is discussed below.

### 3.3 Gq activation of PV cells induces phasic firing, which reduces gamma oscillations and facilitates fear expression

**Figure 1:**
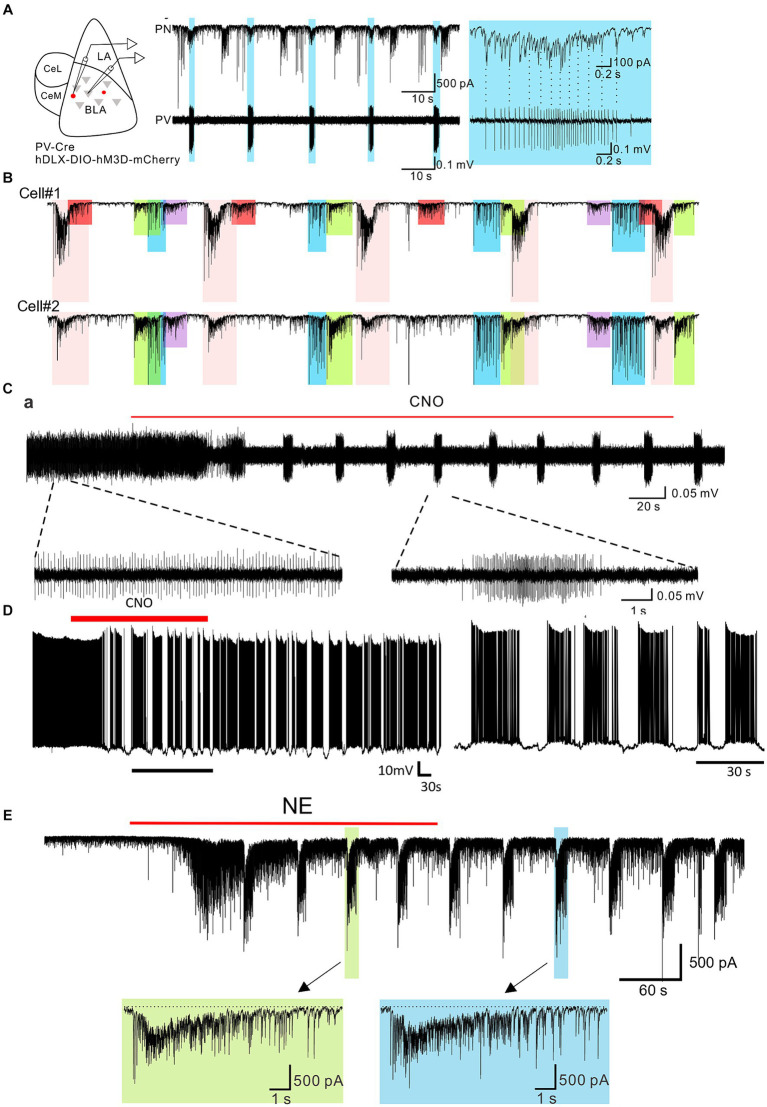
Burst generation in BLA parvalbumin basket cells and principal neurons by neuromodulatory activation. **(A)** Left: schematic diagram of paired recording of principal neurons and PV neurons in the BLA. Middle: Continuous whole-cell recording of a principal neuron (PN) and loose-seal recording of a PV-expressing neuron (PV) during CNO activation of Gq-DREADDs in PV neurons. Right: Expanded recording of the IPSC burst in the principal neuron and the spike burst in the PV neuron shown by the asterisk. Shading shows the spike and IPSC bursts that are synchronized between the two cells. **(B)** Loose-seal patch clamp recording from a Gq-DREADD-transduced PV neuron showing the transition from tonic spiking to phasic spiking with the application of CNO. Lower traces are expansions of the designated periods of the upper trace. **(C)** Whole-cell current clamp recording of the voltage response in a principal neuron to Gq-DREADD activation of PV basket cells showing the phasic activity induced by bursting inputs from PV neuron(s). **(D)** Paired whole-cell voltage clamp recordings from two principal neurons during DREADD activation of PV neurons. Color-coded shading represents IPSC bursts that are synchronized between the two neurons. The different colored bursts were generated by different presynaptic PV neurons projecting to both cells. The red-shaded bursts were seen in only Cell #1, suggesting that the presynaptic PV neuron projected only to Cell #1 and not to Cell #2. **(E)** Whole-cell voltage clamp recording of the IPSC response to NE in a principal neuron. NE elicited an initial, single burst of IPSCs that was mediated by a CCK neuron input and a prolonged train of repetitive IPSC bursts that was mediated by a PV neuron input. The bottom trace is an expansion of one of the PV neuron-mediated bursts (designated by the arrow). Modified from Fu et al. (2022).

The PV-positive basket cells comprise 20% of the total number of interneurons in the BLA (Viktória et al., 2021) and have been shown to control spike generation in principal cells, modulate BLA network activity, and gate the process of fear learning (Wolff et al., 2014; Davis et al., 2017; Veres et al., 2017; Ozawa et al., 2020; Antonoudiou et al., 2022; Fu et al., 2022). In addition to activation by glutamatergic synaptic inputs, the PV cells in the BLA are also regulated by neuromodulators. We recently showed that selective activation of Gq-coupled designer receptors exclusively activated by designer drugs (hM3D DREADDs) in the PV cells induced a stereotypic bursting pattern of action potentials in the PV interneurons and a corresponding bursting pattern of inhibitory postsynaptic currents (IPSCs) in postsynaptic principal cells in the BLA (Figures 1A,B) (Fu et al., 2022). The IPSC bursts typically lasted for a few seconds and were repeated every 20–30 s, and continued for 10’s of minutes after washout of the designer drug. The patterned IPSC bursts were generated specifically by Gq activation, and not continuous depolarization of the PV cells, as sustained optogenetic stimulation of PV cells generated tonic, not phasic, IPSCs in the BLA principal cells. The patterned IPSC bursting input to spontaneously active postsynaptic neurons converted a tonic pattern to a phasic pattern of firing in the BLA principal neurons (Figure 1C). Consistent with each PV cell innervating the perisomatic region of hundreds of postsynaptic principal cells in the BLA (Vereczki et al., 2016), 68% of the recorded IPSC bursts were found to be synchronized between neighboring pairs of BLA principal cells (Figure 1D), suggesting a potential role of Gq neuromodulation of PV interneurons in the regulation of BLA oscillatory states.

The Gq-mediated bursting pattern in PV basket cells raised the possibility that other neuromodulators can also stimulate the same activity through GPCRs coupled to Gq signaling pathways. Supporting this idea, application of norepinephrine (NE) (Figure 1E) and serotonin (5-HT) both induced repetitive bursts of IPSCs in the BLA principal cells via activation of Gq-coupled receptors. This effect was mediated by Gq activation in PV interneurons as it was eliminated by a selective Gq inhibitor YM-254890 and by blockade of PV neuron transmission with a P/Q-type calcium channel blocker (Fu et al., 2022). Furthermore, NE- and 5-HT-driven IPSC bursts were abolished by blocking α1A and 5-HT2A receptors, respectively, which are expressed in the BLA PV cells and conventionally activate Gq signaling (Hadcock and Malbon, 1993; Roth et al., 1998; Jiang et al., 2009; Fu et al., 2022). In the frontal cortex, activation of M1 muscarinic receptors known to couple to Gq also stimulated phasic bursts of IPSCs (Kondo and Kawaguchi, 2001). Therefore, different neuromodulatory systems recruited during emotional arousal can converge on the same intracellular signaling pathway and stimulate similar patterns of neural activity to achieve the desired brain state. That different neurotransmitters acting at different receptors can arrive at very similar endpoints by activating a common intracellular signaling pathway was surprising.

In the cortex and hippocampus, tonic PV neuron activity is critical for the generation of gamma oscillations, which was demonstrated by the activity of PV cells phase locked to the gamma cycle and by optogenetic activation and inhibition of PV neuron activity (Hájos et al., 2004; Bartos et al., 2007; Cardin et al., 2009; Sohal et al., 2009; Antonoudiou et al., 2022). As a corollary, disruption of tonic PV activity interferes with the generation of gamma oscillations. In brain slices, Gq activation in the PV cells transformed tonic PV spiking activity into phasic bursting, which in turn drove a phasic pattern of spiking activity in the postsynaptic BLA principal cells (Figures 1B–D) (Fu et al., 2022). Consistent with PV neuron tonic activity being instrumental in the generation of gamma oscillations, the transformation of PV activity to phasic firing by Gq activation via either chemogenetic or α1 adrenoreceptor activation reduced gamma power in the BLA both in *ex vivo* brain slices and *in vivo* (Fu et al., 2022).

Suppression of fast gamma oscillations has been shown to correlate with conditioned fear expression (Stujenske et al., 2014). Consistent with this, chemogenetic or α1A adrenergic receptor activation of Gq signaling in BLA PV interneurons potentiated the expression of conditioned fear (Fu et al., 2022). Thus, Gq signaling-induced bursting output of the PV interneurons serves as a novel convergent cellular mechanism for different neuromodulators to gate the switching of neural network and behavioral states during emotional arousal.

### 3.4 Gq activation in CCK basket cells induces rhythmic activity and enhances theta oscillation

Due to their expression of receptors for multiple neuromodulators, CCK basket cells have been proposed to integrate signals from subcortical neuromodulatory systems and to encode emotional states (Freund and Katona, 2007; Armstrong and Soltesz, 2012; Cea-del Rio et al., 2012). In contrast to the phasic IPSC bursting activity generated by α1A adrenoreceptor activation of Gq signaling in presynaptic PV cells, NE induced a tonic IPSC pattern in the BLA principal cells that was mediated by activation of presynaptic CCK basket cells since it was blocked by N-type calcium channel blockers and by CB1 receptor activation (Fu et al., 2022). Both the CCK and PV basket cell-mediated IPSCs were detected in the same BLA principal neurons, suggesting the activities of BLA principal cells are modulated by NE via both CCK and PV basket cells. The CCK basket cell-mediated IPSCs showed a stereotyped rhythmic frequency of ~4 Hz that was abolished by an α1A adrenoreceptor antagonist and by a selective Gq inhibitor, and was absent in the α1A receptor knockout mouse (Fu et al., 2022). Since CCK basket cell-mediated tonic IPSCs and PV basket cell-mediated phasic IPSCs were both stimulated by NE activation of α1A adrenoreceptors, the two distinct patterns of NE-induced IPSC outputs were likely generated by recruiting different G-protein signaling pathways or ion channel combinations in the PV and CCK basket cells. Overall, activation of Gq signaling through α1A adrenoreceptors stimulated distinct types of IPSC outputs from the two basket cell types in the BLA, with the CB1-expressing CCK-positive basket cells generating rhythmic IPSCs at theta frequency and the PV-positive basket cells generating a phasic bursting pattern of IPSCs. Interestingly, the bursting frequency of the PV basket cells corresponded to an infra-slow rhythm, although PV cell activation of infra-slow network oscillations in the BLA was not detected (Fu et al., 2022). These observations suggest that the CCK and PV basket cells differentially tune the oscillatory states of the BLA upon neuromodulatory activation and that the unique properties of the downstream Gq signaling in each cell type give rise to cell type-specific output patterns that further delineate the functional dichotomy of CCK and PV basket cells.

As with the Gq-mediated IPSC bursts in the PV interneurons, the CCK-type rhythmic IPSCs are not uniquely induced by a specific neuromodulator, but rather are generalizable to other neuromodulators that engage Gq signaling. One well-characterized exemplar neuromodulator that activates CCK basket cells is acetylcholine (ACh). The general cholinergic receptor agonist carbachol has been used for decades to induce theta oscillations in the hippocampus, which is largely mediated by inhibitory synaptic transmission and is sensitive to CB1 receptor activation and the muscarinic receptor antagonist atropine, suggesting the involvement of neuromodulatory activation of CCK basket cells in the theta rhythm generation (Reich et al., 2005; Alger et al., 2014; Nagode et al., 2014). Furthermore, optogenetic stimulation of cholinergic fibers induced rhythmic IPSCs in the theta range in both the hippocampus and the BLA (Nagode et al., 2011; Bratsch-Prince et al., 2023). Those IPSCs were also sensitive to CB1 receptor activation, by either DSI or CB1 agonist, and to optogenetic inhibition of CCK interneurons, confirming the involvement of CCK basket cells, as well as to a Gq-coupled cholinergic receptor (M3) antagonist (Nagode et al., 2011; Bratsch-Prince et al., 2023). At the circuit level, cholinergic activation of the CCK basket cells increases theta oscillatory power in both the BLA and hippocampus (Nagode et al., 2011; Vandecasteele et al., 2014; Bratsch-Prince et al., 2023). These results collectively suggest that the CCK basket cells generate distinct inhibitory outputs in response to Gq stimulation to modulate the theta oscillation of neural networks.

One major technical challenge to selectively labeling and manipulating the CCK basket cells to understand their functions stems from the heterogeneity of CCK-positive neurons. Since CCK or its preprohormone is also expressed at low levels in principal neurons (Taniguchi et al., 2011), an intersectional strategy combining CCK-Cre mouse line with either DLX viruses or a DLX5/6-FLPO mouse line allows specific labeling of the CCK interneurons (Dimidschstein et al., 2016; Vogel et al., 2016; Rovira-Esteban et al., 2019). However, the CCK interneurons labelled via these intersectional strategies showed poor selectivity for the CB1-positive CCK basket cells, as PV basket cells, axo-axonic cells, and neurogliaform cells were also labeled (Rovira-Esteban et al., 2019; Grieco et al., 2023). Although optogenetic activation of CCK interneurons overall facilitated fear extinction (Rovira-Esteban et al., 2019), the function of CCK basket cells in fear processing still remains elusive. Benefiting from transcriptomic analyses of cortical interneurons, the Scng-Flpo transgenic mouse line was engineered to label the CCK basket cells with superior specificity (Dudok et al., 2021). Future studies utilizing the Scng-Flpo line or other methods to selectively manipulate CCK basket cells in the BLA will provide invaluable insights into the roles of CCK basket cells and neuromodulation-mediated theta oscillation in fear memory formation.

## 4 Conclusion

**Figure 2:**
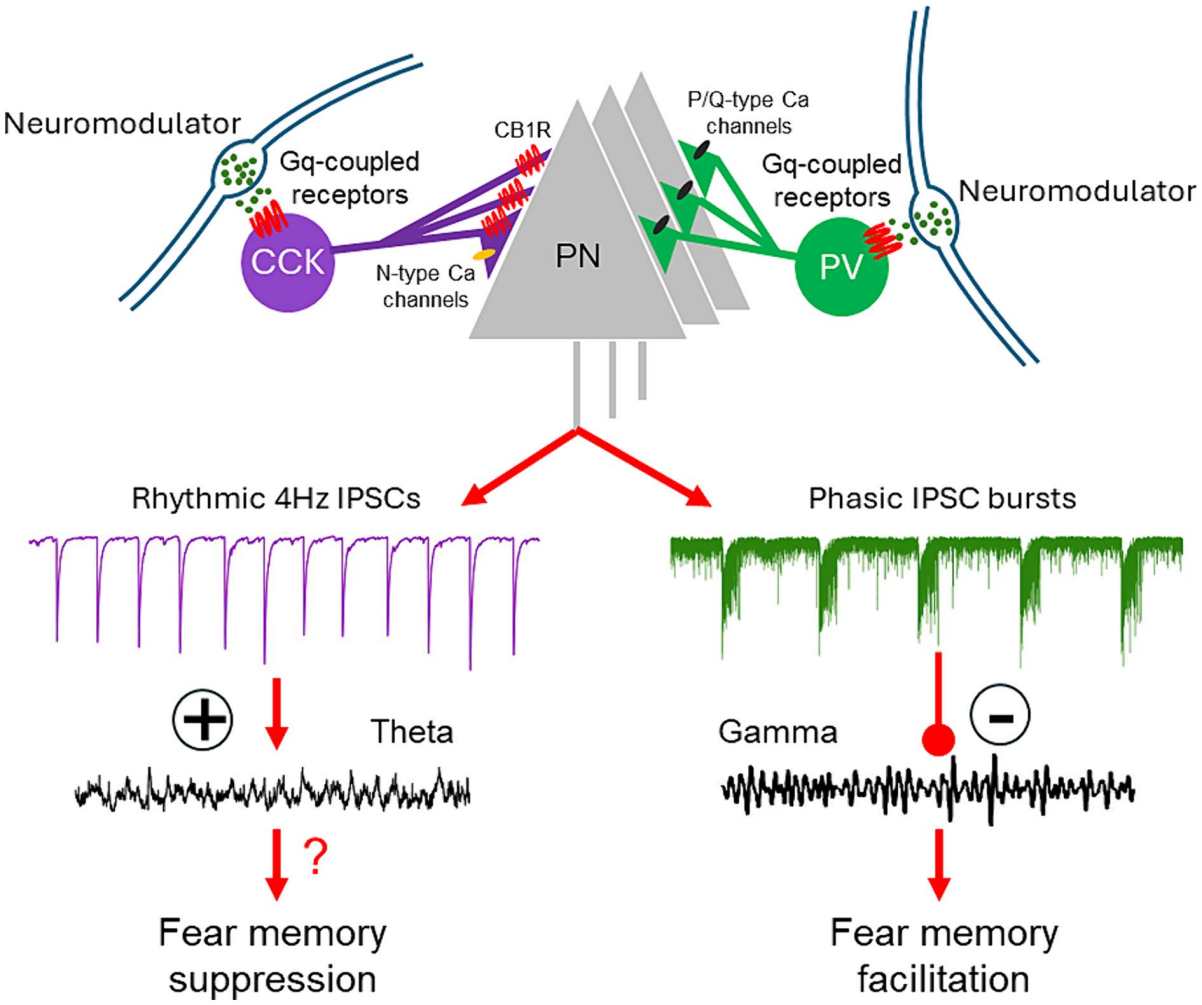
Model of regulation of BLA network activity and fear learning via neuromodulation of CCK and PV basket cells. Activation of Gq-coupled receptors on CCK and PV basket cells stimulates CB1R-sensitive, rhythmic IPSCs and repetitive, high-frequency bursts of IPSCs in the BLA principal cells. These distinct patterns of synaptic input, respectively, enhance theta oscillations and suppress gamma oscillations in the BLA network. The Gq neuromodulatory activation of PV interneurons facilitates fear memory formation; the effect of Gq neuromodulatory activation of CCK basket cells on fear learning remains to be determined.

Neuromodulatory Gq activation in perisomatic PV-positive and CCK-positive basket cells stimulates cell type-specific patterns of inhibitory postsynaptic currents in BLA principal neurons (Figure 2). These distinct patterns of activation are not unique to one neurotransmitter or one receptor subtype, but rather represent a generalizable cellular mechanism for neuromodulation by multiple neurotransmitters to tune the BLA oscillatory network state and behavioral expression of fear. The unique patterns of activity induced by Gq activation in the PV and CCK basket cells further define the capacity of these two inhibitory basket cell types to differentially control the population output of the BLA, and point to the importance of the different patterns of perisomatic inhibitory inputs in determining BLA output. Gq-coupled DREADDs have been used extensively as a tool to test the causal role of activation of neurons of interest in behavior and physiology, assuming a general increase of the excitability and neural activity of the infected cells. However, caution needs to be exercised in interpreting the results as Gq neuromodulation of the cells may introduce a pattern of neural network activation beyond a simple enhancement of activity, which may reveal the function of the activated circuits within different operational modes.

## Author contributions

XF: Conceptualization, Formal analysis, Writing – original draft, Writing – review & editing. JT: Conceptualization, Funding acquisition, Project administration, Resources, Supervision, Writing – review & editing.
